# Mitochondrial 2,4-dienoyl-CoA reductase (Decr) deficiency and impairment of thermogenesis in mouse brown adipose tissue

**DOI:** 10.1038/s41598-019-48562-x

**Published:** 2019-08-19

**Authors:** Anne M. Mäkelä, Esa Hohtola, Ilkka J. Miinalainen, Joonas A. Autio, Werner Schmitz, Kalle J. Niemi, J. Kalervo Hiltunen, Kaija J. Autio

**Affiliations:** 1 Faculty of Biochemistry and Molecular Medicine, University of Oulu, Oulu, Finland; 2 Department of Ecology and Genetics, University of Oulu, Oulu, Finland; 3 BCO Imaging core facilities, University of Oulu, Oulu, Finland; 4 Center for Biosystems Dynamics Research, RIKEN, Kobe, Japan; 5 Medical Research Center, University of Oulu and Oulu University Hospital, Oulu, Finland; 6 University of Würzburg, Würzburg, Germany

**Keywords:** Fat metabolism, Energy metabolism

## Abstract

A large number of studies have demonstrated significance of polyunsaturated fatty acids (PUFAs) for human health. However, many aspects on signals translating PUFA-sensing into body homeostasis have remained enigmatic. To shed light on PUFA physiology, we have generated a mouse line defective in mitochondrial dienoyl-CoA reductase (Decr), which is a key enzyme required for β-oxidation of PUFAs. Previously, we have shown that these mice, whose oxidation of saturated fatty acid is intact but break-down of unsaturated fatty acids is blunted, develop severe hypoglycemia during metabolic stresses and fatal hypothermia upon acute cold challenge. In the current work, indirect calorimetry and thermography suggested that cold intolerance of *Decr*^−/−^ mice is due to failure in maintaining appropriate heat production at least partly due to failure of brown adipose tissue (BAT) thermogenesis. Magnetic resonance imaging, electron microscopy, mass spectrometry and biochemical analysis showed attenuation in activation of lipolysis despite of functional NE-signaling and inappropriate expression of genes contributing to thermogenesis in iBAT when the *Decr*^−/−^ mice were exposed to cold. We hypothesize that the failure in turning on BAT thermogenesis occurs due to accumulation of unsaturated long-chain fatty acids or their metabolites in *Decr*^−/−^ mice BAT suppressing down-stream propagation of NE-signaling.

## Introduction

Core body temperature is crucial for survival of homeothermic animals in terms of keeping physical conditions amenable for maintenance of homeostasis and physiological activities. In addition to basal metabolism and mechanical work, mammals possess two other processes to enhance thermogenesis on demand; shivering thermogenesis in muscles and non-shivering thermogenesis in brown adipose tissue (BAT). BAT is a tissue capable of heat generation by uncoupling mitochondrial respiration from ATP generation through inner mitochondrial membrane residing uncoupling protein 1 (UCP1). UCP1 dissipates energy charge of transmembrane chemiosmostic force (H^+^ gradient) as heat^1^. Non-shivering thermogenesis in BAT is strictly controlled by the sympathetic nervous system, which conveys the stimulus of cold by releasing norepinephrine (NE) at adrenergic synapses in BAT. NE binds to β_3_-adrenergic receptors, leading to an increase in intracellular cAMP concentration, followed by the initiation of intracellular signaling cascades required for thermogenesis; increased oxygen consumption, lipolysis and activation of gene expression. Free fatty acids released from white adipose tissue and taken up from circulation or intracellular lipid droplets are fueling thermogenesis and also acting as activators of UCP1.

**Figure 1:**
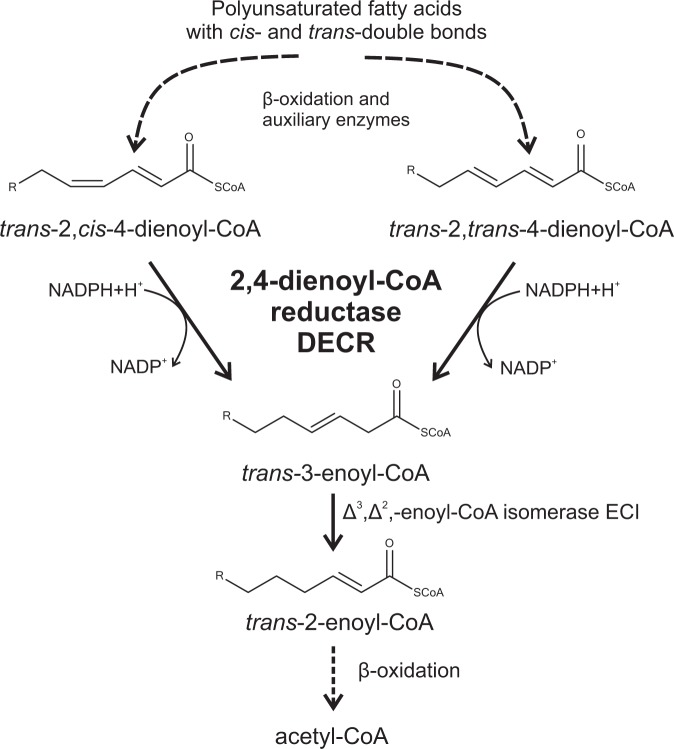
Degradation of polyunsaturated fatty acids in mitochondria.

Polyunsaturated fatty acids (PUFAs) play multiple roles in maintenance of tissue homeostasis and human health. PUFAs and their metabolites regulate gene expression through various mechanisms including interactions with transcription factors, but also via causing changes in intracellular calcium level, membrane composition and eicosanoid production. In addition to regulation of gene expression, PUFAs are involved in post-translational regulation of protein abundance by regulating protein turn-over and stability^2^. Independent of either nutritional or endogenous metabolic origin of PUFAs in the body, their double bonds are mainly *cis*-bonds in either odd or even numbered position. Concerning catabolism of PUFAs, β-oxidation is quantitatively the major pathway, but *trans*-2-enoyl-CoA esters are the only unsaturated intermediates accepted by β-oxidation enzymes as substrates. Therefore, β-oxidation of unsaturated fatty acids calls for contribution of auxiliary enzymes. One among them is 2,4-dienoyl-CoA reductase (Decr) found both in mitochondria and peroxisomes, encoded by separate genes^3^. Decr catalyses the NADPH-dependent reduction of Δ^2^,Δ^4^-dienoyl-CoA esters to *trans*-3-enoyl-CoA esters (Fig. 1)^4^.

To study the functional role of mitochondrial Decr in mammals, a knock-out mouse model for Decr deficiency was generated^5^. In the *Decr*^−/−^ model, the most prominent features are the development of fatty liver and hypoglycemia. Furthermore, when the *Decr*^−/−^ mice are exposed to +4 °C, after a short shivering period their body temperature drops and in 3–4 hours they become lethargic (hypothermia). In contrast to other defects of fatty acid oxidation, where phenotypes can be explained by failure in mitochondrial ATP synthesis, absence of the Decr activity does not inhibit oxidation of straight chain fatty acids as demonstrated by turning on hepatic ketogenesis as a response to fasting. The aim of this work was to shed light on the cold intolerance of *Decr*-deficient mice. Indirect calorimetry studies showed reduced oxygen consumption with concomitant decrease in body temperature in *Decr*^−/−^ mice when ambient temperature was decreasing linearly. The fat content in iBAT did not decrease in *Decr*^−/−^ mice during the fasting, although the total body fat content decreased similar to the wild-type mice. The data showed that although NE-firing is on and there are ample fuels available upon exposure of *Decr*^−/−^ mice to the cold, the signal is not translated to appropriate thermogenesis in BAT of *Decr*^−/−^. The findings demonstrate the essence of functional break-down of PUFAs for adaptation to metabolic stresses in mice.

## Results

### Indirect calorimetry

**Figure 2:**
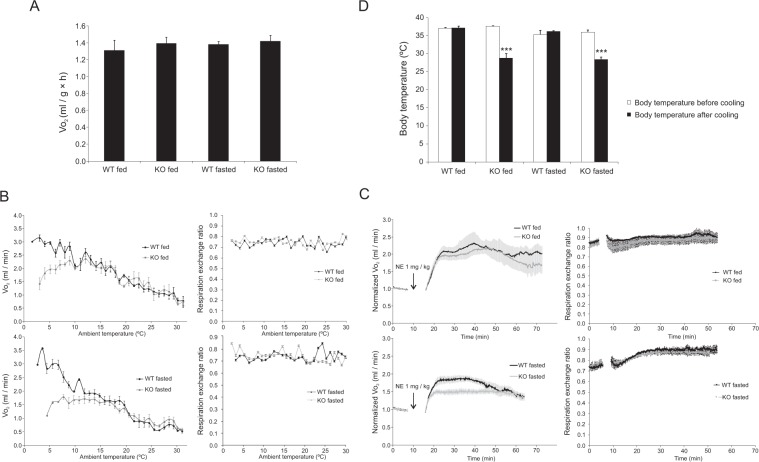
Indirect calorimetry. (**A**) Basal metabolic rate (BMR) at +31 °C of *Decr*^−/−^ mice (KO) and wild type mice (WT) with or without prior 18 h-fasting indicated as milliliters of oxygen consumed per hour per gram of body weight. (**B**) Oxygen consumption (*V*O_2_) and respiratory exchange ratio RER of WT and KO mice upon 6 h linear cooling exposure measured by indirect calorimetry. Upper graphs: oxygen consumption and respiratory exchange ratio of mice under fed state. Lower graphs: oxygen consumption and respiratory exchange ratio of mice after fasting of 18 hours. (**C**) Oxygen consumption and respiratory exchange ratio of WT and KO mice after NE-induction of BAT thermogenesis (1 mg/kg body weight, s.c.) at +31 °C. NE-injection was given after obtaining a 10-min stable basal value. The mean *V*O_2_ from this period was set to 1 to calculate the relative increase in *V*O_2._ After placing the mice back to the chambers, oxygen consumption was measured for 40 minutes until the experiment was terminated. Upper graphs: oxygen consumption and respiratory exchange ration of fed mice. Lower graphs: oxygen consumption and respiratory exchange ratio of mice with prior fasting of 23 hours. (**D**) Body temperature of WT and KO mice at the beginning and at the end of cold exposure of indirect calorimetry. Statistically significant differences between wild type and *Decr*^−/−^ mice are indicated by asterisks (***p < 0.001). Values are shown as group (n = 4) means ± SE.

Indirect calorimetry enables estimation of heat produced by the animal via calculations based on oxygen consumption *V*O_2_. Mice with or without prior fasting were placed into individual chambers inside of a temperature controlled cabinet. At the beginning, an ambient temperature of +31 °C was maintained until the BMR of the animals was stabilized. There were no significant differences in the BMR between groups. Under these thermoneutral conditions the oxygen consumptions of the non-fasted and fasted *Decr*^−/−^ mice were 1.39 O_2_ (ml/h × g^−1 body weight^) and 1.42 O_2_ (ml/h × g^−1 body weight^), respectively. The corresponding values for the wild type fed and fasted mice consumption were 1.31 O_2_ (ml/h × g^−1 body weight^) and 1.38 O_2_ (ml/h × g^−1 body weight^) (Fig. 2A).

After obtaining BMR, the ambient temperature was linearly lowered from +31 °C to +3 °C during a time course of six hours (4.8 °C/hr) to force the mice to increase their metabolic rate in order to maintain a normal body temperature. Under these conditions, oxygen consumption should be enhanced due to the activation of shivering and BAT thermogenesis. Wild type mice in fed state increased their oxygen consumption throughout the cooling phase, reaching an average maximum consumption of 3 ml/min (> 3-fold) per mouse at the end of the experiment. The maximum oxygen intake of fed *Decr*^−/−^ mice was on average 2.3 ml/min per mouse, which was reached at the ambient temperature of +14 °C in three hours followed by a plateau until the oxygen consumption started to decline (Fig. 2B). Wild type mice were able to increase their metabolic rate during cold exposure despite prior fasting. By contrast, the maximum oxygen intake of the *Decr*^−/−^ mice after fasting was reduced to 1.75 ml/min already at +17 °C and continued to decline upon further cooling (Fig. 2B). There were no differences in respiratory exchange ratios between the wild type and *Decr*^−/−^ mice in fed condition or after fasting during the cooling experiment (Fig. 2B).

In a complementary experiment, cold exposure was mimicked by injecting anesthetized mice with NE, the natural stimulator of BAT thermogenesis. The fed mice of both genotypes responded first to NE-stimulation by increasing their oxygen consumption 2-fold. Thereafter the difference between the genotypes became evident. At 60 minutes after the NE-injection the *Decr*^−/−^ mice started to show a reduced oxygen intake compared to the wild type mice. Fasting blunted the response of both types, but significantly more in *Decr*^−/−^ mice. The initial response of the fasted *Decr*^−/−^ mice was similar to the response of the wild type animals, but after 20 min when the rate of oxygen consumption had reached 1.55 ml/min on average per mouse it started to decline and leveled off to 1.4 ml/min, and remained below the wild type mice until the end of the experiment (Fig. 2C). There were no differences in respiratory exchange ratios between the wild type and *Decr*^−/−^ mice in fed or fasted condition after NE injection (Fig. 2C).

The body temperature of mice was measured at the beginning and after terminating the indirect calorimetry study, when the ambient temperature was linearly lowered from +31 °C to +3 °C during a time course of six hours. Both fed and fasted wild type mice maintained their body temperature throughout the experiment, whereas *Decr*^−/−^ mice developed severe hypothermia (Fig. 2D), which, however, was not aggravated by fasting (final temperatures 28.8 vs, 28.4 °C, respectively).

### Interscapular brown adipose tissue (iBAT)

**Figure 3:**
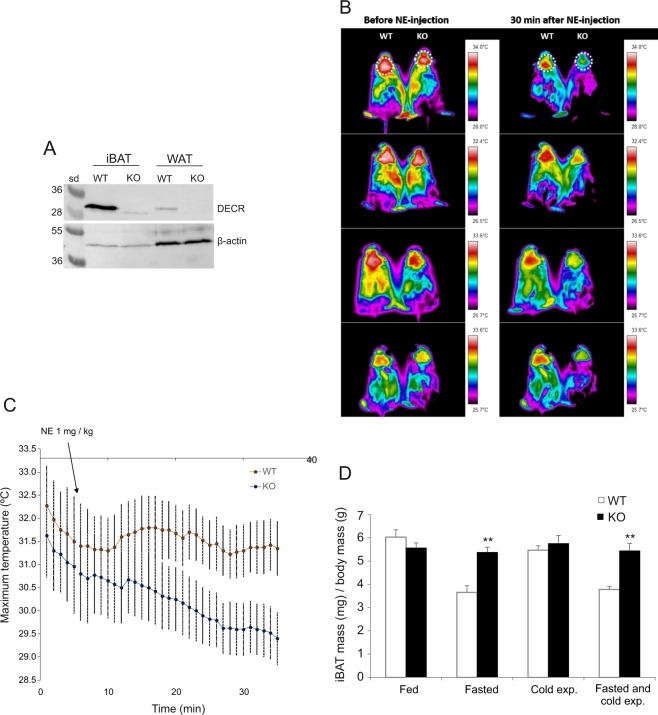
Heat production by interscapular brown adipose tissue (iBAT). (**A**) Western blot analysis of iBAT and WAT tissue homogenates by using antibody against rat DECR showing presence or absence of the 33 kDa band corresponding to DECR in wild type (WT) and *Decr*^−/−^ (KO) mice, respectively. β-actin was used as a loading control. (**B**) Thermography of heat formation on the surface above iBAT was studied from fasted wild type (WT) and *Decr*^−/−^ mice (KO) pairwise (WT on the left, KO on the right) using infrared imaging. Mice were anesthetized and injected subcutaneously with NE (1 mg/kg body weight). Imaging of the heat formation on the surface above iBAT (circled area) was continued 30 min after the NE-injection. The general decreasing trend in both groups is due to the anesthesia. (**C**) Maximum surface temperatures were calculated using image analysis software. Values are expressed as means of ± SE of four mouse pairs. (**D**) iBAT mass index was analyzed from WT and KO mice from four treatment-groups: (i) normal feeding, no fasting, (ii) fasting (24 hours), (iii) cold exposure (3 hours) and iv) fasting (24 hours) including cold exposure (3 hours). Values are expressed as group (n = 9) means ± SE. Statistically significant differences are indicated by asterisks (*p < 0.05, **p < 0.01).

The results obtained from the indirect calorimetry experiments suggested a defect in the BAT thermogenesis in *Decr*^−/−^ mice, and thus we zoomed our studies toward the biggest single depot of BAT, the iBAT. First we confirmed by western blotting that there are no mitochondrial DECR protein (size ~33 kDa) expressed in iBAT or white adipose tissue (WAT) in *Decr*^−/−^ mice (Fig. 3A). Our antibody against rat DECR^6^ detects also a peroxisomal 2,4-dienoyl-CoA reductase that is 31 kDa protein in mice^7^ seen as a faint band in iBAT sample from *Decr*^−/−^ mice. Next, heat production in iBAT was analyzed by infrared imaging. Mice were fasted for 24 hours, anesthetized, injected with NE, and imaged pairwise (wild type and a *Decr*^−/−^ mouse) with an infrared camera for 30 minutes (Fig. 3B). Maximum surface temperatures of the skin above the iBAT were analyzed using image analysis software (Fig. 3C). The results showed reduced heat formation above iBAT of fasted *Decr*^−/−^ mice compared to fasted wild type mice.

The iBAT (mg × 1000) and body weight (g) mass index was analyzed from altogether 70 age-matched male and female mice that were divided in four treatment-groups: (i) chow feeding, no fasting, (ii) fasting (24 hours); (iii) cold exposure (3 hours) and (iv) fasting (24 hours) with a cold exposure during the last 3 hours. After treatment the mice were sacrificed and iBAT was dissected, carefully separated from white adipose tissue (WAT) and weighed. In the fed state, the indexes of wild type and *Decr*^−/−^ mice were comparable the values being 6.0 and 5.6, respectively. After fasting iBAT mass index in the wild type mice was reduced to 3.7 whereas the index was unchanged in *Decr*^−/−^ mice. Three hours of cold exposure had an effect on iBAT mass index in wild type mice (reduction from 6.0 to 5.5), but in contrast no change in *Decr*^−/−^ mice during this observation period. After fasting combined with cold exposure wild type mice again had a lower iBAT mass index (3.8) compared to *Decr*^−/−^ mice (5.4) (Fig. 3D).

**Figure 4:**
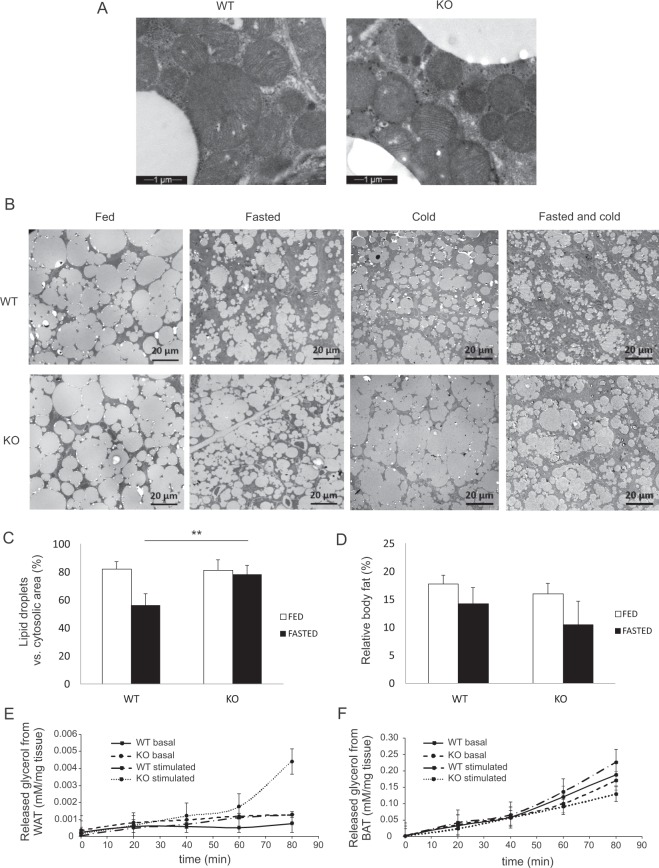
Electron microscopy analysis of iBAT, whole body magnetic resonance imaging and lipolysis in adipose tissues. (**A**) Representative EM images from iBAT of wild type (WT) and *Decr*^−/−^ (KO) mice on fed stage (images were taken with 13500 x magnification). (**B**) Representative EM images from iBAT of WT and KO mice on fed and fasted stage and after cold exposure (images were taken with 890 x magnification). (**C**) Phase analysis of lipid droplets vs. cytosolic area on EM images in fed and fasted state. The values are shown as group (n = 10 images) means of ± SE. Statistically significant differences between WT and KO mice are indicated by asterisks (**p < 0.01). (**D**) Relative body fat/water content in WT and KO mice before and after fasting as quantified using magnetic resonance imaging. Data are presented as group means ± SE. (n = 6). *Ex vivo* lipolysis rates in (**E**) WAT and (**F**) BAT under basal and isoproterenol-stimulated conditions (n =4–5).

To visualize the iBAT in the fed mice, electron microscopic (EM) analysis were carried out. Under fed conditions, the EM-images of iBAT showed similar tissue and cellular organization in both the wild type and *Decr*^−/−^ mice. The gross analysis of micrographs showed that typically to BAT, cells were in contact with capillaries and nerve fibers. The cells contained numerous lipid droplets of various sizes and were rich in mitochondria with well-organized and dense cristae (Fig. 4A). After 24 hours of fasting, number and sizes of lipid droplets were decreased in wild-type mice, but these changes were not so prominent in *Decr*^−/−^ mice iBAT (Fig. 4B). Similarly, after the cold exposure for three hours the size of lipid droplets in wild type mice iBAT were decreased while *Decr*^−/−^ mice iBAT lipid droplet were unchanged. After fasting and cold exposure the size of lipid droplets in *Decr*^−/−^ mice iBAT were decreased, but also in this case the changes were more prominent in wild type mouse iBAT. To quantitate the observed changes due to fasting, the area occupied by lipid droplets versus cytosolic area was calculated from EM-images captured with a CCD camera using phase analysis (iTEM software). The phase analysis revealed that the percentage of the lipid droplet occupation area was reduced from 82% to 56% in the wild type brown adipocytes during the fasting, whereas the change in the iBAT of the *Decr*^−/−^ mice after fasting was not significant (Fig. 4C).

In order to determine whether the reduced mobilization of triacylglycerol storages during fasting is a general defect in lipolysis in adipose tissues of *Decr*^−/−^ mice, whole body relative fat/water content was quantified by using magnetic resonance imaging before and after fasting. Since body fat is mostly composed of WAT and brown fat represents only a small fraction of total fat, the BAT contribution could be omitted from the whole body fat calculation and thus the results are presented here as “WAT”. In fed state, the relative fat/water content was 17.7% ± 1.5 in wild type mice and 16.0% ± 1.9 knockout mice, indicating similar fat content across groups. Upon fasting, fat content was reduced to 14.2% ± 2.9 and 10.5% ± 4.2 in wild type group and in fasted *Decr*^−/−^ mice, respectively (Fig. 4D). These data point that the overall lipolysis was functional in WAT both in wild type and *Decr*^−/−^ mice.

To assess directly the lipolysis in WAT and iBAT in wild type and *Decr*^−/−^ mice, the release of glycerol from adipocytes isolated from WAT and from iBAT explants was measured. The samples were collected in basal condition and 0, 20, 40, 60 and 80 minutes after stimulus of isoproterenol. Release of glycerol was slightly higher from white adipocytes in *Decr*^−/−^ mice than wild type mice and stimulation with isoproterenol increased the glycerol release in both genotypes (Fig. 4E). *Ex vivo* glycerol release from iBAT was slightly lower in *Decr*^−/−^ mice than wild type mice in basal condition. Stimulation in wild type mouse iBAT caused increase in glycerol release, but in *Decr*^−/−^ mice iBAT isoproterenol stimulation led to decreased glycerol release during the time course (Fig. 4F). These results indicate that lipolysis in WAT is functional in *Decr*^−/−^ mice, but in iBAT during the time course lipolysis is inhibited.

**Figure 5:**
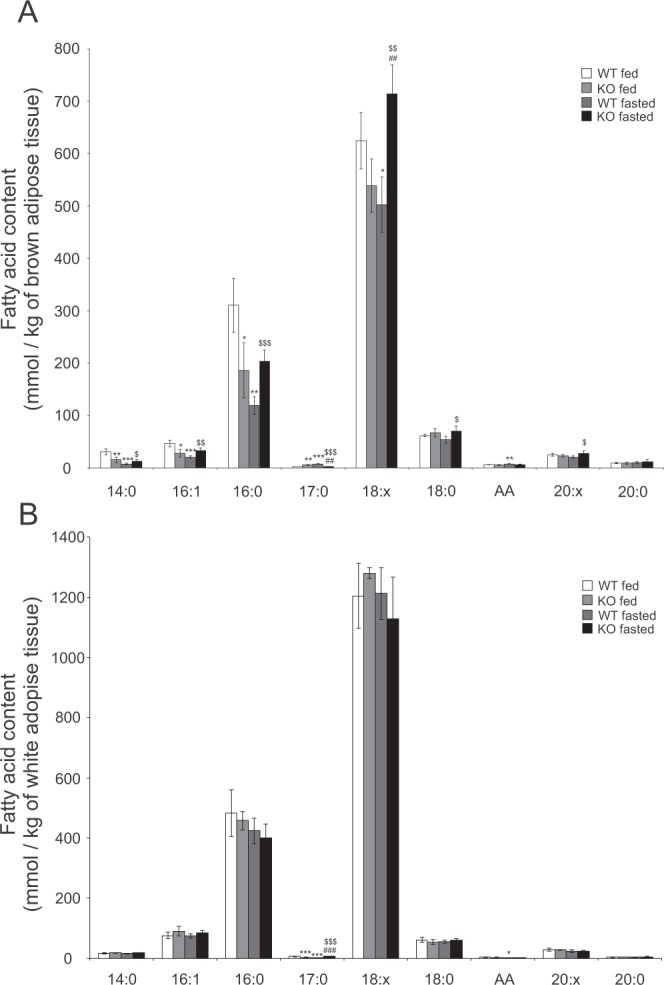
MS analysis of iBAT and WAT fatty acids. Total triacylglycerol bound fatty acid content and composition in brown adipose tissue (**A**) and white adipose tissue (**B**) of wild type (WT) and *Decr*^−/−^ (KO) mice in fed and fasted stages were analyzed by mass spectrometry. WT fed mice versus *Decr*^−/−^ fed mice or fasted wt *p < 0.05, **p < 0.01, and ***p < 0.001; *Decr*^−/−^ fed mice versus fasted *Decr*^−/−^ mice ^#^p < 0.05, ^##^p < 0.01, and ^*##*#^p < 0^.^001; and fasted wt versus fasted *Decr*^−/−^ mice ^$^p < 0.05, ^$$^p < 0.01, and ^$$$^p < 0.001. AA = arachidonic acid.

The fatty acid composition and the degree of fatty acid saturation were analyzed from iBAT and WAT samples by mass spectrometry. In iBAT the main fatty acids are unsaturated octadecenoic and hexadecenoid acids and saturated hexadecanoic and octadecanoic acids. In fed state the amount of 16 carbons long hexadecanoic and hexadecenoic acid is decreased in *Decr*^−/−^ mice iBAT compared to wild type counterparts. During the fasting the amount of fatty acids in BAT of wild type mice is decreased, but in *Decr*^−/−^ mice BAT the octadecenoic acid content is elevated and the amounts of hexadecanoic, octadecanoic and hexedecenoic acids remain unchanged compared to fed stage (Fig. 5A). When comparing fatty acid levels of wild-type and *Decr*^−/−^ mice after fasting, *Decr*^−/−^ mice have higher levels of both unsaturated as well as saturated fatty acids compared to wild-type. In WAT the fatty acid composition is almost identical to BAT the main fatty acid species being unsaturated octadecenoic and hexadecenoic acids and saturated hexadecanoic and octadecanoic acids. Although the overall body WAT declined in both animal groups upon fasting as a sign of mobilization of triacylglycerol storages, the amount and quality of fatty acid species per weight of tissue did not change in WAT from either wild type or *Decr*^−/−^ mice (Fig. 5B).

Functionality of mitochondrial β-oxidation in iBAT was tested from isolated iBAT mitochondria by using palmitoylcarnitine as a substrate. The rate of mitochondrial β-oxidation was monitored as ferricyanide reduction in uncoupled, rotenone and cyanide poisoned mitochondria in the presence of added oxaloacetate and cytochrome c. The estimated rates of iBAT β-oxidation were 3.2 ± 1.5 µmol/min per mg protein in wild type mice (n = 5) and 3.8 ± 2.3 µmol/min per mg protein in *Decr*^−/−^ mice (n = 5) showing that central β-oxidation pathway of saturated fatty acids in iBAT mitochondria is functional in Decr-deficient mouse.

### Decr deficiency alters parameters involved in thermogenesis

**Figure 6:**
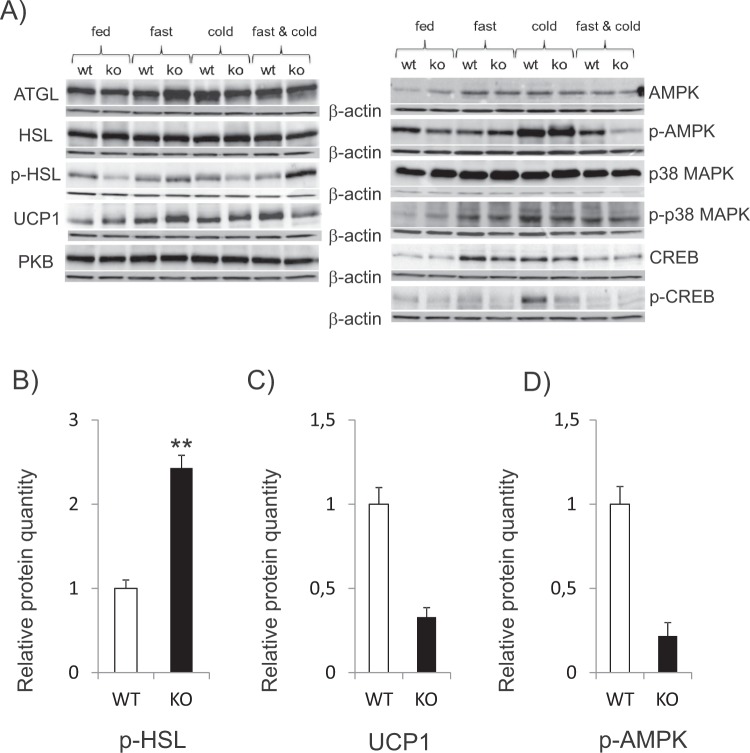
Western blot analysis. Representative immunoblots of proteins involved in regulatory pathways of lipolysis and thermogenesis in iBAT of wild type (WT) and *Decr*^−/−^ (KO) mice. Test groups: (i) no fasting, no cold exposure, (ii) fasting (24 hours), no cold exposure, (iii) cold exposure (3 hours) and (iv) fasting (24 hours) including cold exposure (3 hours). (**A**) Factors involved in lipolysis cascade; ATGL, HSL, p-HSL, and UCP1 as the key factor in thermogenesis and kinases involved in intracellular signalling cascades; PKB, AMPK, p38 MAPK and transcription factor CREB. β-Actin served as a loading control. Relative protein quantities of p-HSL (**B**), UCP1 (**C**) and p-AMPK (**D**). Values are presented as group (n = 5) means ± SE from three technical repeats.

Since the data described above suggested that thermogenesis in iBAT of Decr-deficient mice does not response to cold in a proper manner, the cellular factors contributing to control lipolysis and thermogenesis were analyzed. For this purpose, iBAT proteins were extracted from mice of four treatment groups as described above. The total protein levels of adipose triacylglycerol lipase (ATGL) or hormone sensitive lipase (HSL) were not changed, but phosphorylation of HSL was affected (Fig. 6A). Fasting and cold exposure together were accompanied by an increased p-HSL level in *Decr*^−/−^ mice (relative quantity 2.4) than in wild type mice (relative quantity 1) (Fig. 6B). In paradox, the critical protein in terms of heat production, UCP1, was decreased in the iBAT of fasted and cold exposed *Decr*^−/−^ mice (relative quantity 0.33) (Fig. 6C). This may partially explain the reduced thermogenesis of *Decr*^−/−^ mice.

Several other components involved in intracellular signaling pathways participating in lipolysis and thermogenesis were also studied. There were no differences between genotypes or treatment groups on protein amounts of AMP-activated protein kinase (AMPK). The cold exposure activated the phosphorylation of AMPK in both wild type and *Decr*^−/−^ mice BAT, but the level of p-AMPK was down regulated in the fasted and cold exposed *Decr*^−/−^ mice (relative quantity 0.22) when compared to the wild type (Fig. 6D). There were no differences between genotypes or treatment groups on protein amounts of protein kinase B (PKB/Akt), p38 mitogen-activated protein kinase (p38 MAPK) and phosphorylated p38 MAPK, the transcription factor cAMP-responsive element binding protein (CREB) or phosphorylated CREB (p-CREB) (Fig. 6A).

### Reduced expression of thermogenic genes in Decr^−/−^ iBAT

**Figure 7:**
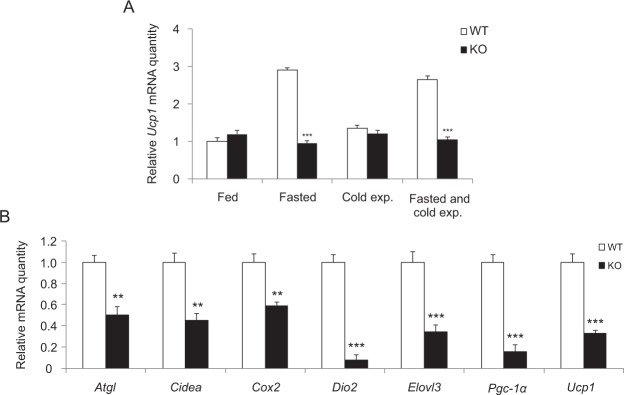
Quantitative RT-PCR analysis of thermogenic gene expression. (**A**) Expression of *Ucp1* mRNA in iBAT of wild type (WT) and *Decr*^−/−^ (KO) mice. Treatment groups were (i) no fasting, no cold exposure, (ii) fasting (24 hours), no cold exposure, (iii) cold exposure (3 hours) and (iv) fasting (24 hours) including cold exposure (3 hours). Fold change of mRNA expression of fed WT mice was set as a reference and has a value of 1 on a linear scale. Mouse β-actin and GAPDH were used as endogenous controls to which sample values were normalized. Values are presented as group (n = 5) means ± SE from three individual measurements. Statistically significant differences between WT and KO mice are indicated by asterisks (***p < 0.001). (**B**) Gene expression of thermogenic genes *Atgl, Cidea, Cox2, Dio2, Elovl3, Pgc-1α* and *Ucp1* in iBAT of fasted (24 h) and cold exposed (3 h) wild type (WT) and *Decr*^−/−^ (KO) mice. Fold change of mRNA expression of fasted and cold exposed WT mice was set as a reference and has a value of 1 on a linear scale. Mouse β-actin and GAPDH were used as endogenous controls to which sample values were normalized. Values are expressed as group (n = 5) means ± SE from three individual measurements. Statistically significant differences between WT and KO mice are indicated by asterisks (**p < 0.01, ***p < 0.001).

Enhancement of heat production in BAT is often accompanied by induced expression of a set of genes encoding factors involved in thermogenesis. Quantitative RT-PCR analysis was applied to study gene expression profiles in iBAT upon stimulation of thermogenesis. Mouse groups were as previously described: (i) no fasting, no cold exposure, (ii) fasting (24 h), (iii) cold exposure (3 h) and (iv) fasting with cold exposure (24 h including 3h-cold exposure). Fasting alone activates thermogenesis, which could be clearly seen in fasted wild type mice but not in *Decr*^−/−^ mice. For example *Ucp1* expression was 2.9-fold induced in the iBAT of fasted wild type mice and 2.7-fold induced in the BAT of fasted and cold exposed wild type mice. In contrast, *Decr*^−/−^ mice showed no clear transcriptional response (Fig. 7A).

Typically with the genes analyzed from iBAT, the most pronounced differences between genotypes were seen in the group of 24 hours of fasting combined with a 3-hour cold exposure. Compared to the wild type, the *Decr*^−/−^ mice show an inability to respond to fasting conditions by up regulation of expression of *Atgl, Cidea*, *Cox2*, *Dio2*, *Elovl3*, *Pgc-1α* and *Ucp1*, which normally are induced upon thermogenesis activation (Fig. 7B). The lowest expression levels in addition to *Ucp1* were detected with the key thermogenic genes *Dio2* (fold change 0.08) and *Pgc-1α* (fold change 0.16) when compared to fasted and cold exposed wild type mice. Strong down regulation of *Dio2* is a firm indication of error in the activation of thermogenesis in BAT of *Decr*^−/−^ mice.

### Fibroblast growth factor 21(Fgf21) expression is up-regulated in Decr^−/−^ mice

**Figure 8:**
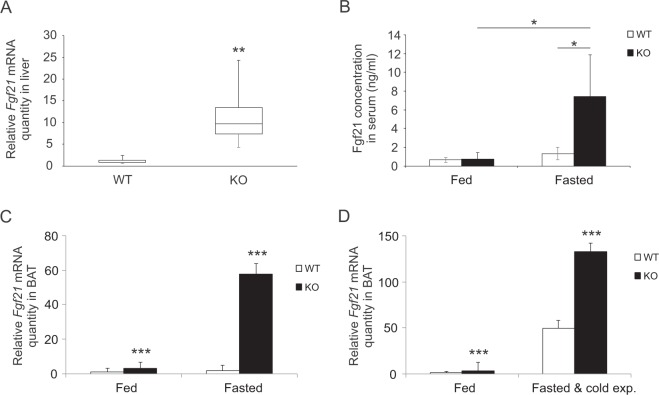
*Fgf21* expression levels in liver and iBAT and FGF21 concentration in circulation. (**A**) Expression of *Fgf21* mRNA in liver after 24 hours of fasting in wild type (WT) and *Decr*^−/−^ (KO) mice. Box whisker plot shows the data from four WT and five KO samples. The p-value is 0.0003. (**B**) Concentration of FGF21 in serum of wild type (WT) and *Decr*^−/−^ (KO) mice in fed and fasted stage (n = 5). Statistically significant differences are indicated by asterisk (*p < 0.05). (**C**) *Fgf21* expression in iBAT of WT and KO mice under fed stage and after fasting. (**D**) *Fgf21* expression in iBAT of WT and KO mice under fed stage and after fasting and cold exposure. Fed WT *Fgf21*expression was set as a reference and has a value of 1 on the linear scale. Statistically significant difference between WT and KO mice are indicated by asterisks (***p < 0.001). Values are expressed as group (n = 5) means ± SE. Mouse β-actin and GAPDH were used as endogenous controls to which sample values were normalized.

Fibroblast growth factor 21 (FGF21) is an endocrine factor with very robust favourable effects on glucose and lipid metabolism in mice^8^. It is mainly expressed in liver, but in mouse also in WAT and BAT and cold exposure and adrenergic signaling induce FGF21 expression in adipose tissues^9,10^. In *Decr*^−/−^ mice liver after 24 hours of fasting the expression of *Fgf21* was upregulated around ten times when compared to wild type mouse hepatocytes after fasting (Fig. 8A). In serum there were no difference in concentrations of Fgf21 in fed stage between wild type and *Decr*^−/−^ mice, but after fasting the concentration of Fgf21in circulation was markedly increased in Decr-deficient mouse compared to fasted wild type animal or *Decr*^−/−^ mice in fed stage (Fig. 8B). Expression of *Fgf21* was highly up regulated in BAT of fasted as well as in fasted and cold exposed *Decr*^−/−^ mice, but also in fasted and cold exposed wild type mice. Fasting induced *Fgf21* expression in BAT by 3-fold in wild type and by 58-fold in *Decr*^−/−^ mice (Fig. 8C). Fasting combined with cold exposure induced *Fgf21* expression by 50-fold in wild type mice BAT, and 133-fold in *Decr*^−/−^ mice BAT (Fig. 8D).

## Discussion

Deficiency of the mitochondrial Decr leaves the central β-oxidation spiral intact and thus allows saturated fatty acids to serve as substrates for mitochondrial respiratory metabolism, whereas mitochondrial β-oxidation of (poly)unsaturated fatty acids is damped^5^. *Decr*^−/−^ mice displayed visually observed muscle shivering during cold exposure. In spite of this, the mice developed hypothermia. Following this observation, an additional source of heat, non-shivering thermogenesis in BAT was taken as the target of further investigation. The obtained findings showed that (i) Decr-deficiency leads to decline in body core temperature in cold at least partly due to failure in turning on non-shivering thermogenesis in BAT, (ii) Decr-deficiency leads to impaired activation of lipolysis in BAT and (iii) Decr-deficiency leads to mis-regulated expression of the key thermogenic genes in BAT. These disturbances can raise as outcomes of non-functional NE-firing, disrupted intracellular signaling, short of substrate supply for mitochondrial respiration and failure in uncoupling of mitochondrial respiration.

Exposure of animals to cold triggers adrenergic firing in BAT, release of NE and binding of NE to β3-adrenergic receptors that leads to intracellular signaling cascade ultimately turning on non-shivering thermogenesis. As a part of this cascade, the activated protein kinase A (PKA) will phosphorylate a number of intracellular regulatory proteins, among them perlipin (PLIN) and HSL, which control intracellular lipolysis^11,12^. As an indicator for NE-firing, phosphorylation of HSL was observed to occur in the *Decr*^−/−^ mice upon fasting and exposing animals to cold. This observation speaks against the idea that failure in maintaining body temperature is due to dysfunctional adrenergic firing in BAT. Functional lipolysis has been considered as the key step in non-shivering thermogenesis in BAT, where released fatty acids act as fuel and also as activators of UCP1^13^. In Decr-deficient mouse under fasting and cold stress both the weight of iBAT and the area occupied by lipid droplets in EM pictures were increased suggesting failure in lipolysis in iBAT. Direct measurement of lipolysis from iBAT explants showed decreased lipolysis in *Decr*^−/−^ mice compare to wild type during the time course.

However, it is unlikely that this explains the cold intolerance of *Decr*^−/−^ animals as recent publications using BAT-specific knock-out models of *Atgl* or *Abhd5* have challenged the role of lipolysis in thermogenesis in BAT^14,15^. These data indicated that ATGL-mediated lipolysis in not prerequisite for cold-induced non-shivering thermogenesis and fasting-induced lipolysis in WAT (and heart and liver) is a sufficient supplier of fuel for non-shivering thermogenesis and ultimately to maintain body temperature^14–16^. Our experiments showed that in *Decr*^−/−^ mice lipolysis in WAT is functional. The concentrations of glycerol released from white adipocytes were slightly higher in Decr-deficient mouse than wild types and in both genotypes the stimulation of lipolysis enhanced the glycerol level. Previously we have demonstrated that the concentration of nonesterified fatty acids increased from 0.5 to 1.0 mM with a concomitant increase of 3-hydroxybutyric acid from 0.13 to 1.11 mM in serum in *Decr*^−/−^ mice during 24 h fasting^5^ indicating mobilization of triacylgycerol storages. Additionally, MRI analysis demonstrated a decrease in body fat mass, presenting mainly WAT, in Decr-deficient mice similarly as in wild type animals as a response to fasting.

The observation that lipolysis is turned on in both wild type WAT and BAT upon stress, prompts the question why lipolysis is activated in Decr-deficient WAT but not in BAT. Concerning regulation of lipolysis on lipid droplets, under basal conditions activation of ATGL is suppressed by binding of ABHD5 to PLIN. Adipocyte lipolysis via NE- and cAMP-dependent PKA activation is initiated by PLIN phosphorylation that triggers the release of ABHD5 from the complex to activate ATGL and to a recruitment of phosphorylated HSL^11,12,17^. Lipolysis can be either suppressed or promoted by ligands^12^, which interact directly with ABHD5 and either cause its release from PLIN or further promote the binding. Among the ligands identified are long chain acyl-CoAs which can bind ABHD5 and enhance its binding to PLIN. Binding to ABHD5 and thus suppression of lipolysis was observed with palmitic acid (C16:0) as well as with hexadecenoic acid (oleic acid, C18:1) in a concentration-dependent manner^12^. During fasting, contents of C16:0 and C18:X decreased in wild type mouse BAT, but were actually increased in the Decr-deficient BAT (Fig. 5). In WAT, the levels of C16:0 and C18:X did not change due to fasting or mouse genotype.

The increased C18:X content in BAT can be explained in terms of non-functional Decr, the activity that is required for β-oxidation of unsaturated fatty acids with double bonds either odd- or even-numbered positions^4,18^. Mitochondrial β-oxidation of saturated fatty acids is functional in Decr-deficient BAT, so elevated levels of saturated fatty acids in *Decr*^−/−^ mice iBAT are due to non-functional lipolysis leading to accumulation of saturated fatty acids in BAT. One potential mechanism could be accumulation of unsaturated fatty acids in BAT inhibiting the lipolysis. Curiously, similar to the wild type animals, the NE-injection in fasted *Decr*^−/−^ mice resulted to an initial burst of the oxygen consumption (Fig. 2C) showing that non-shivering thermogenesis was initiated. However, the burst was truncated in few minutes, a phenomenon that could be due to mobilization and subsequent accumulation of inhibitory metabolites of unsaturated fatty acids.

It has been shown that adipocyte AMPK is required in activation of BAT thermogenesis by sympathetic drive and maintaining the mitochondrial homeostasis in BAT^19^ as well as AMPK phosphorylates ATGL and HSL^20,21^. However, Mottillo *et al*.^19^ showed that lack of adipocyte AMPK does not affect lipolysis and thus the role of AMPK in regulation of lipolysis is controversial. Concerning AMPK in the BAT of *Decr*^−/−^ mice, in agreement with the general rule and as it happens in the fed control animals, the proportion of phosphorylated AMPK increased when the mice were exposed to cold. As a paradox, lipolysis in *Decr*^−/−^ BAT was not proceeding under these conditions. Of note, the correlation between p-AMPK and p-HSL does not hold if the *Decr*^−/−^ mice that were subjected to acute fasting and cold, conditions that were accompanied by a decrease in p-AMPK, but HSL was highly phosphorylated. Taken together, these data suggest existence of other factors than the low p-AMPK leading to the non-responsive NE-signaling under these conditions.

UCP1 has been regarded as responsible factor for heat production in BAT and long chain fatty acids as activators of UCP1^22^. In Decr-deficient BAT upregulation of *Ucp1* expression due to fasting for 24 hours does not work (Fig. 7A). In our experiments, a 3-hour cold exposure in fed state did not increase the expression of *Ucp1* in wild type or *Decr*^−/−^ BAT. The initial housing temperature was +21 °C, and in this condition brown adipocytes have already achieved full differentiation and thus no induction of UCP1 after cold exposure can be seen^23^. After fasting and cold exposure both expression and protein level of UCP1 were decreased in *Decr*^−/−^ BAT compared to wild type as well as expression of multiple thermogenic genes (Figs 6 and 7).

Mechanisms to activate thermogenesis beyond UCP1, like creatine-dependent substrate cycling and calcium-dependent ATP hydrolysis, have been described recently (for review, see^24^). Creatine-dependent substrate cycling has been studied by using mouse models where the rate-limiting enzyme of creatine synthesis, glycine amidotransferase or creatine transporter has been knocked out in adipose tissue^25,26^. In both models defect in creatine-dependent substrate cycling leads to obesity. Furthermore, there were no change in Ucp1 expression in BAT in creatine deciency, while fasting or fasting with cold exposure caused a decrease in Ucp1 levels in *Decr*^−/−^ BAT. Sarcolipin (Sln) is a regulator of the sarco/endoplasmic reticulum Ca^2+^-ATPase (SERCA) pump and appears to uncouple calcium transport from ATP hydrolysis by SERCA. Sln-knock out mice are cold-intolerant and gain more weight on high-fat diet compared wild type animals^27^. There are no difference in body weight between wild type and *Decr*^−/−^ mice and to due to phenotype differencies, it is unlikely that in deficiency in creatine-dependent substrate cycling or calcium-dependent ATP hydrolysis are behind the phenotype seen in *Decr*^−/−^ mice. Thus, cold intolerance observed in Decr-deficient mice can be due to defective activation of UCP1.

FGF21 is an liver-derived hepatokine and in mouse it is expressed also in BAT. The upregulation of *Fgf21* expression in response to cold exposure, fasting and also increased fatty acid load has been reported^28–30^. FGF21 has been implicated in browning of adipose tissue^31,32^ and FGF21 treatment in mouse have been shown to cause increased expression of thermogenic genes and genes involved in lipolysis^33^. In Decr-deficient mouse the expression of *Fgf21* in liver after fasting was ten times higher compared to wild type animals and in serum the Fgf21 concentrations were elevated in *Decr*^−/−^ mice after fasting. In BAT, the *Fgf21* expression was found to be higher in *Decr*^−/−^ mice under basal conditions (3-fold compared to wild type) and fasting with cold exposure amplified the *Fgf21* expression in *Decr*^−/−^ mice. It is possible that Fgf21 expression is induced via lipid sensor GPR120. GPR120 is a G-protein-coupled receptor that bind unsaturated long fatty acids and their derivatives^34^. Activation of GPR120 induces release of Fgf21 in BAT^35^. Interestingly, it has been shown that GPR120 suppresses adipose tissue lipolysis^36^.

Cold intolerance is a feature observed in several mouse models with deficiencies directly involved in mitochondrial fatty acid β-oxidation or processes feeding acyl-groups into this pathway. Mouse models presenting enzyme deficiencies in this category are listed in Table 1. These models include mice deficient of enzymes catalysing the first step of fatty acid β-oxidation, the acyl-CoA dehydrogenases SCAD, MCAD, LCAD or VLCAD, and mice deficient of hydroxyacyl-CoA-dehydrogenases M/SCHAD and LCHAD, enzymes of the third step of β-oxidation. Similarly, mice deficient of either subunit of mitochondrial trifunctional protein (MTP) are unable to maintain their body temperature, if exposed to cold. Many other phenotypes are also shared between these mouse models, including lipid accumulation in liver and hypoglycaemia (Table 1). The phenotype of *Cpt2a*^−/−^ mice who are deficient in transport of long-chain fatty acyl-groups into the mitochondria, resemble in many respects the phenotype of *Decr*^−/−^ mice. In particular, these mice were refractory to and fail to upregulate thermogenic genes in response to adrenergic agonist-induced stimulation^37,38^. Also, mice deficient of the deacetylase SIRT3 present a similar phenotype as *Decr*^−/−^ mice; fatty liver, hypoglycemia and severe cold intolerance^39^. SIRT3 deficiency leads to increased acetylation of LCAD thus reducing LCAD activity, which is supposed to lead to accumulation of LCFAs. Based on this observation, it could be speculated that accumulating LCFAs in the BAT of *Sirt3*^−/−^ mice might cause suppression of lipolysis and lead to impaired thermogenesis.

**Table 1:** Mouse models with a phenotype including cold intolerance (see text for further details).

Mouse model	Enzyme	Metabolic function	Mouse phenotype	References
*Scad* ^−/−^	Short-chain acyl-CoA dehydrogenase	Mitochondrial β-oxidation	Fasting and cold intolerance, fatty liver and kidney	^50^
*Mcad* ^−/−^	Medium-chain acyl-CoA dehydrogenase	Mitochondrial β-oxidation	Lower blood glucose, slightly elevated serum FFA concentration upon fasting, cold intolerance	^51, 52^
*Lcad* ^−/−^	Long-chain acyl-CoA dehydrogenase	Mitochondrial β-oxidation	Sudden death, gestational loss, fatty change of liver and heart, cold intolerance	^53, 54^
*Vlcad* ^−/−^	Very long-chain acyl-CoA dehydrogenase	Mitochondrial β-oxidation	Milder version of *Lcad*^−/−^, hepatic and myopathic phenotypes	^55, 56^
*M/Schad* ^−/−^	Medium- and short-chain L-3 hydroxyacyl-CoA dehydrogenase	Mitochondrial β-oxidation	Fasting and cold intolerance, fatty liver and kidney	^57^
*Lchad* ^−/−^	Long-chain 3-hydroxyacyl-CoA dehydrogenase	Mitochondrial β-oxidation	Hypoketotic hypoglycaemia, hepatopathy, cardiomyopathy, developmental delay	^58– 60^
*Eci1*^−/−^/*Eci2*^−/−^	Enoyl-CoA isomerase	Mitochondrial β-oxidation	Mild phenotype, normal ketogenesis, trend to lower blood glucose, elevated blood C12:1	^61^
*Decr* ^−/−^	2,4-dienoyl-CoA reductase	Mitochondrial β-oxidation	Fatty liver, severe hypoglycaemia and cold intolerance	^5^
*Acsl1* ^−/−^	Acyl-CoA synthetase-1	Activation of long-chain fatty acids to acyl-CoAs that are directed towards mitochondrial β-oxidation	Reduced FA oxidation and O_2_-consumption, hypoglycaemia, cold intolerance	^41^
*Atgl* ^−/−^	Adipose triacylglycerol lipase	Lipolysis	Reduced lipolysis, cold intolerance, *Pparα* and *Ucp1* expression down regulated	^62, 63^
*CD36* ^−/−^	Fatty acid translocase	Facilitation of long-chain fatty acid transport on membrane	Decreased FA uptake, hypoglycaemia, cold intolerance, gene expression in BAT down regulated	^64^
*Fabp4/5* ^−/−^	Fatty acid binding protein 4 and 5	Facilitation of lipid transport to specific compartments in the cells	Hypoglycemia, cold intolerance, depletion of intracellular lipids in BAT and muscle	^65^
*Fabp3* ^−/−^	Fatty acid binding protein 3	Facilitation of lipid transport to specific compartments in the cells	Severe cold intolerance, induced expression of oxidative genes, failure in oxidizing endogenous fatty acids in BAT	^44^
*Sirt3* ^−/−^	Mitochondrial NAD+-dependent deacetylase	Control of acetylation status of many enzymes involving in energy metabolism	Increased acetylation of LCAD, lipid accumulation in the heart, cold intolerance	^39^
*Tfe3* ^−/−^	Transcription factor E2	Regulation of lipid metabolism in adipose tissue	Decreased ATGL mRNA level, decreased lipolysis in BAT, cold intolerant	^66^
*Lcn2* ^−/−^	lipocalin 2	Innate immune response	Hyperglycemia, cold intolerance, reduced oxidative capacity in BAT and muscles	^45^

Mouse models showing cold intolerance, but with a defect elsewhere in the fatty acid metabolism than directly within β-oxidation, include mice deficient of ACSL1, ATGL, CD36, FABP4/5 and FABP3. An adipose specific acyl-CoA synthetase-1 deficient (*Acsl1*^*A*−/−^) mouse model is cold intolerant and shows reduced adipose FA oxidation. ACSL1 functions in directing FAs towards β-oxidation. Similarly to *Decr*^−/−^ mice, they respond to acute cold challenge with adrenergic signalling in BAT, but are not able to increase oxygen consumption upon β_3_-adrenergic stimulation^40,41^. Lack of ATGL prevents lipolysis and CD36 deficiency interferes with fatty acid uptake into the cell. FABP4/5 deficiency likely leads to a defect in intracellular fatty acid trafficking. The proposed cause for cold intolerance is commonly suggested to be energy deficiency due to lack of fuel substrates in BAT^42,43^. A somewhat different conclusion can be drawn from a mouse model deficient of the heart-type fatty acid-binding protein, FABP3, which seems to be essential for fatty acid oxidation and cold tolerance. The severely cold intolerant *Fabp3*^−/−^ mice show impaired oxidation of endogenous fatty acids in BAT^44^, making this model an interesting analogy to the *Decr*^−/−^ model. Hypoglycemia as such is known to be a thermogenesis-suppressing condition. It could thus be assumed that the severe hypoglycaemia of *Decr*^−/−^ mice would simply lead to failure in thermogenesis. However, the data from indirect calorimetry with fed mice argues against this theory, since they are not hypoglycemic, but still develop hypothermia during the linear cooling phase. Furthermore, the mouse model deficient of lipocalin 2 (*Lcn2*^−/−^), a protein involved in the innate immune response, also suffer from cold intolerance, but in contrast to *Decr*^−/−^ mice, these mice show increased hepatic gluconeogenesis and hyperglycemia^45^.

### Conclusion and summary

Under standard conditions, accumulated PUFAs are shielded in lipid droplets, and *Decr*^−/−^ mice are symptomless substantiated by the functional peroxisomal β-oxidation that is fully equipped for metabolism of PUFAs. Upon fasting or acute cold stress, triacylglycerols in lipid droplets in WAT are subject to lipolysis and the *Decr*^−/−^ mice who are unable to breakdown unsaturated fatty acids are exposed to a load of unesterified PUFAs. The Decr-deficient mitochondria in BAT are unable to cope with this PUFA load. We hypothesize that accumulating PUFAs and their metabolites have pleiotropic effects on BAT activities including inhibition of thermogenesis, lipolysis and dysregulation of thermogenic gene expression although sympathetic outflow towards BAT. Although *Decr*^−/−^ mice present a general knock-out mouse model, their phenotype displays tissue specify towards liver^5^ and BAT. The results show the importance of functional oxidative metabolism of unsaturated fatty acids for balanced physiological responses toward stresses. We have initiated experiments with isolated cells and BAT mitochondria designed to depicture the key inhibitory PUFA derived drivers of non-shivering thermogenesis.

## Experimental

### Animal studies

The *Decr* null mutant (*Decr*^−/−^) mouse line was generated as described by Miinalainen and co-workers^5^. The *Decr*-knockout was maintained in the C57BL/6 background and C57BL/6 mice were used as wild type controls. Mice were housed in the Laboratory Animal Centre of the University of Oulu. Mouse maintenance conditions included a normal 12 hour lighting period (07:00–19:00) and unrestricted access to standard chow and water. In most experiments, three to six months old male mice were used, except iBAT mass index analysis where both sexes were used. In *ex vivo* lipolysis and β-oxidation measurements both sexes at age of 16–17 months. No difference between sexes were detected. For experiments, the mice were fasted as indicated for either 12 or for 24 hours, with free access to water. When needed for experimental purposes, mice were anesthetized with Hypnorm-Dormicum-solution, containing 0.063 mg/kg (body weight) fentanyl citrate, 20 mg/kg (body weight) fluanisone and 1 mg/kg (body weight) midazolam. Dosage used was 0.08 ml/10 g of body weight (s.c.). For activation of brown adipose tissue mice were injected with norepinephrine 1 mg/kg of body weight (s.c.). In acute cold exposure experiments, mice were housed individually and exposed to +4 °C for a maximum of 3 hours. Animals were handled in strict accordance with good animal practice, and animal experiments were conducted according to the EU directive 2010/63/EU and Finnish legislation. Animal experiments were evaluated and approved by the Finnish national committee for the protection of animals (license numbers ESAVI/8707/04.10.07/2014 and ESAVI/1116/04.10.03/2011).

### Indirect calorimetry

Mice were placed in individual chambers inside of a temperature controlled cabinet. The relatively small space of the chambers led the mice to calm down and stay still to avoid the interference caused by extra muscle movement. At the beginning of the experiment, an ambient temperature of +31 °C was maintained until all mice reached a stable basal metabolic rate (BMR). Thereafter the mice were subjected to cooling by linearly lowering the ambient temperature from +31 °C to +3 °C during 6 hours. In another series of experiments, acute cold exposure was mimicked by NE-injection. Mice were anesthetized, placed into the chambers for 10 min for BMR measurement, injected (sc.) with NE (1 mg/kg body weight) and placed back into the chambers for 40 min. The rectal temperature was measured with a custom-made 26-gayge copper-constantan thermocouple before and after placing the mice into the chambers. Oxygen consumption and carbon dioxide production in both series was measured in an open-flow respirometer system. Pressurized outside air was scrubbed of carbon dioxide and water using soda lime and silica gel, respectively, and passed to a Perspex metabolic chamber at a flow rate of 400 ml/min (STP). The airflow was regulated by Bronkhorst High-Tech mass-flow controllers (Bronkhorst High-Tech, Ruurlo, the Netherlands). For oxygen analysis, a sample (ca. 100 ml/min) of outlet air was obtained using a sub-sample pump (SS4, Sable Systems International, Las Vegas, NV, USA), scrubbed again of carbon dioxide and water, and directed to an oxygen analyzer (Model 1440, Servomex, UK). For carbon dioxide, a similar subsample was drawn, the gas was dried in a Peltier cooler a,d then directed to a carbon dioxide analyzer (Model 1440, Servomex, UK). The sensor voltages were recorded every 10 s, using VeePro software (Agilent Technologies, Santa Clara, CA, USA). Reference gas was sampled every 20 min in long term experiments. Oxygen consumption *V*O_2_ was calculated using equations appropriate for the measurement where water vapor and carbon dioxide are removed before analysis, i.e. equation 2 of Hill^46^. Carbon dioxide production *V*CO_2_ was calculated simply as flow times concentration in outlet air. Respiratory exchange ratio was calculated as *V*O_2_/*V*CO_2_.

### Infrared thermography

Pairs (wild type/*Decr*^−/−^) of fasted mice were anesthetized and injected with NE (1 mg/kg, sc.) to induce BAT activation. The maximum temperature of the skin above the interscapular BAT (iBAT) was recorded for 35 min from the images obtained using the ThermaCAM (Researcher 2001, FLIR systems, Wilsonville, OR, USA).

### Transmission electron microscopy analysis

Small pieces dissected from iBAT were fixed in 1% glutaraldehyde 4% formaldehyde mixture in 0.1 M phosphate buffer. They were postfixed in 1% osmium tetroxide, dehydrated in acetone and embedded in Epon LX 112 (Ladd Research Industries, Williston, VT, USA). Thin sections (70 nm) were cut with a Leica Ultracut UCT ultramicrotome, stained in uranyl acetate and lead citrate and examined in a Tecnai G2 Spirit transmission electron microscope (FEI Europe, Eindhoven, The Netherlands) operated at 100 kV. The area occupied by lipid droplets versus the cytosolic area was calculated from images captured with a CCD camera using phase analysis (iTEM software, Olympus Soft Imaging Solutions, Münster, Germany). Phase analysis was done by thresholding the image to two phases based on the pixel grey values.

### Magnetic resonance imaging (MRI) analysis

Fasted and non-fasted mice were sacrificed before MRI. Experiments were performed using a 4.7 T horizontal magnet (Magnex Scientific, Abington, UK) interfaced to a DirectDrive console (Agilent, Palo Alto, CA, USA) in combination with transmit/receive volume RF coil (Rapid Biomedical, Rimpar, Germany). The fat/water imaging sequence was based on selective excitation of the fat and water signals and a 3D gradient-echo for data acquisition^47^. The imaging parameters were repetition time 80 ms, echo time 8.5 ms, flip angle 10°, the offset frequency for fat excitation −702 Hz from water, total imaging time 60 minutes, field-of-view 60 × 60 × 100 mm^3^ and a matrix size of 256 × 128 × 128. Data analysis was performed using Matlab (MathWorks, Natick, MA, USA) and Aedes software (Kuopio, Finland). Images were masked and relative fat-water body content was calculated^48^.

### *Ex vivo* lipolysis

iBAT and epididymal WAT depots were dissected from mice and iBAT (around 10 mg) was put into DMEM (Sigma-Aldrich, St. Loius, MO, USA) with 2% FA-free BSA (Sigma-Aldrich, St. Loius, MO, USA) eWAT was washed and cut into smaller pieces in phosphate-buffered saline (PBS). White adipocytes were released by collagenase (Sigma-Aldrich, St. Loius, MO, USA) in Krebs-Ringer buffer with 2% FA-free BSA for an hour in +37 °C. The white adipocytes were collected by centrifugation for a minute at 400 × g, RT. The cells were washed with Krebs-Ringer buffer with 2% FA-free BSA and centrifuged all together three times. iBAT and eWAT samples were preincubated in DMEM with 2% FA-free BSA at +37 °C for 30 minutes and changed into 150 µl of fresh media. To stimulate the lipolysis, media with 10 µM isoproterenol was used. The adipose tissue depots were incubated at +37 °C and 25 µl sample from media was collected every 20 minutes. The lipolysis in samples was stopped by adding 70% perchloric acid, incubated 15 minutes on ice and neutralized with 1.875 M potassiumcarbonate in 0.5 M triethanolamine. The released glycerol amount in media was determined with glycerol assay kit (Sigma-Aldrich, St. Loius, MO, USA).

### Mitochondrial β-oxidation

Mitochondria from iBAT were isolated by mincing the iBAT into small pieces by razor blade and homogenizing in a Potter homogenizer with a teflon pestle in ice-cold SEM-buffer (250 mM sucrose, 1 mM EDTA, 10 mM Mops, pH 7.2). The tissue homogenate was centrifuged at 8500 × g, 10 minutes at +4 °C and resulting supernatant, containing the floating fat was discarded. The pellet was resuspended in ice-cold SEM-buffer and centrifuged at 800 × g for 10 minutes at +4 °C. The supernatant was collected and centrifuged again at 8500 × g, 10 minutes at +4 °C. The resulting mitochondrial pellet was resuspended in 130 mM KCl, 10 mM HEPES, 0.1 mM EGTA pH 7.2, centrifuged again and finally resuspended in 130 mM KCl, 10 mM HEPES, 0.1 mM EGTA pH 7.2. Mitochondrial β-oxidation rates were measured spectrophotometrically like described^49^. The isolated mitochondrial samples were incubated in presence of 0.5 mM ferricyanide, 1 mM ADP, 1 mM KPi, 1 mM KCN, 0.1 mg/ml cytochrome c and 0.1% bovine serum albumin (essentially fatty acid free) in 130 mM KCl, 10 mM HEPES, 0.1 mM EGTA pH 7.2. 20 µM palmitoylcarnitine was used as a substrate and after addition of 10 mM oxaloacetate and 1 µM carbonyl cyanide p-trifluoromethoxyphenyl hydrazine (FCCP), the rate of ferricyanide reduction was monitored at wavelengths 420 and 470 nm by using Shimadzu UV-3000 spectrophotometer in dual wavelength mode at 20 °C.

### Mass spectrometric (MS) analysis

10 to 20 mg tissue was homogenized in 150 μl chloroform/methanol (6/1, v/v) using a potter equipped with polyurethane pistil. The suspension was diluted with chloroform/methanol (6/1, v/v) to a total volume of 50-times the volume of the homogenized sample fraction. The resulting homogenate was centrifuged (5 min, 18,000 g). 70 μl of supernatant was transferred to a new Eppendorf tube. After the addition of 80 μl chloroform, 20 μl H_2_O and 10 μl 2.5 mM nonadecanoic acid methylester (in chloroform/methanol (1/1, v/v)), the solution was mixed and centrifuged (15 min, 2000 g). The resulting upper phase was discarded and 90 μl of the lower phase were dried at +64 °C under a stream of nitrogen. The residual sample was dissolved in 0.5 ml of 1 M acetylchloride in methanol and heated at +64 °C for 3 hours. The resulting fatty acid methyl esters were extracted twice with 0.5 ml hexane. The extract was dried at +45 °C under a stream of nitrogen. The remainder was dissolved in100 μl hexane and applied to a silica gel TLC plate. The plate was developed in hexane/diethylether (7/3, v/v). Lipids were stained in a reversible process using an iodine cabinet. The fatty acid methylester containing area was scraped off. Fatty acid methyl esters were extracted from the silica powder with 1.5 ml diethylether. The extract was again dried at +45 °C under a stream of nitrogen and the sample was then dissolved in 20 μl dichlorethane. 1 μl of the resulting solution was applied to a GLC column of an ESI-FT-ICR (Fourier-Transform Ion-Cyclotron Resonance Mass Spectrometer, APEX II, Bruker, Bremen, Germany).

### Immunoblotting

Because of small sample sizes, protein samples of iBATs from mice of each treatment and genotype groups (wild type and *Decr*^−/−^) were pooled for Western blot analysis. One pooled sample composed of equal amounts of protein from 5 mice adding up to 50 μg of protein, which was loaded per well in SDS-PAGE and transferred to a 0.2 μm nitrocellulose membrane (Bio-Rad) using a Trans-Blot Turbo-system (Bio-Rad). The membranes were blocked using 5% BSA in TBS 0.1% Tween. Primary antibodies and horseradish peroxidase conjugated secondary antibodies were used to detect proteins of interest (Supplementary Table S1). To analyze the amount of DECR in mouse iBAT and WAT, 15 µg of protein sample from individual mouse iBAT and 30 µg of protein sample from individual mouse WAT were used for a blot and a polyclonal antibody raised against rat 2,4-dienoyl-CoA reductase^6^ was used as a primary antibody. Antibody detection reaction was developed using an Immun-Star^TM^ WesternC^TM^ Chemiluminescent Kit (Bio-Rad), Molecular Imager ChemiDoc^TM^ XRS+ equipment and Image Lab version 3.0 software (Bio-Rad). β-Actin was used as a loading control and for quantification of detected proteins with Image Lab software.

### RNA isolation and quantitative real-time PCR

Total RNA was isolated from mouse brown adipose tissue by using the RNeasy Lipid Tissue Mini Kit. iBAT tissue pieces of 10 mg from 5 mice from each group were pooled for total RNA extraction to represent wild type and *Decr*^−/−^ sample. RNA concentration and quality was analyzed using 2100 Bioanalyzer (Agilent Technologies). Total RNA from mouse liver was extracted by using using Versagene RNA tissue kit (Gentra Systems, Minneapolis, MN, USA). The RevertAid First Strand cDNA Synthesis Kit (Thermo Fisher Scientific, USA) was used to produce cDNA from 1 μg of iBAT RNA or 500 ng of hepatic RNA. The 7500 Real Time PCR System was used with fluorogenic probe-based TaqMan chemistry and the relative standard curve method. TaqMan Gene Expression Assays (Supplementary Table S2) and TaqMan Universal PCR Master Mix (Applied Biosystems, Foster City, CA, USA) were used for iBAT samples throughout the qRT-PCR analyse. For hepatic FGF21, the primers and 5′FAM-labeled probe were designed using Primer Express software (Applied Biosystems) and the sequences available in Genbank and were purchased from Sigma-Genosys (Haverhill, UK). Mouse β-actin and glyceraldehyde-3-phosphate dehydrogenase (GAPDH) were used as endogenous controls to which sample values were normalized. Data was analyzed with DataAssist Software v3.0 (Applied Biosystems).

### Serum FGF21 concentration determination

Concentration of FGF21 in mouse serum was determined by using Quantikine ELISA Mouse/Rat FGF-21 Immunoassay (R&D Systems, Minneapolis, MN, USA).

### Statistical analysis

Results are shown as group means ± standard error (SE). Statistical significance was determined by two-tailed Student´s t-test assuming unequal variance for biological effects with assumed normal distribution. For the change in body temperature in indirect calorimetry, the statistical significance was determined by two-tailed Student's t-test assuming the equal variance, because the values were compared in the same mice.

## Supplementary information

Supplementary information
