# Supplementary material for: Mitochondrial 2,4-dienoyl-CoA reductase (Decr) deficiency and impairment of thermogenesis in mouse brown adipose tissue
Source: Sci Rep. 2019 Aug 19;9:12038. doi: 10.1038/s41598-019-48562-x (PMC6700156; doi:10.1038/s41598-019-48562-x)
Supplement: Supplementary file 1 — Supplementary information [file 41598_2019_48562_MOESM1_ESM.pdf]

SUPPLEMENTARY INFORMATION

Mitochondrial 2,4-dienoyl-CoA reductase (Decr) deficiency and impairment of thermogenesis in mouse brown adipose tissue

Anne M. Mäkelä[1], Esa Hohtola[2], Ilkka J. Miinalainen[3], Joonas A. Autio[4, 5], Werner Schmitz[6], Kalle J. Niemi[1] , J. Kalervo Hiltunen[1] & Kaija J. Autio[1*]

[1]*Faculty of Biochemistry and Molecular Medicine, University of Oulu, Oulu, Finland*

[2]*Department of Ecology and Genetics, University of Oulu, Oulu, Finland*

[3]*BCO Imaging core facilities, University of Oulu, Oulu, Finland*

[4]*Center for Life Science and Technologies, RIKEN, Kobe, Japan*

[5]*Medical Research Center, University of Oulu and Oulu University Hospital, Oulu, Finland*

[6]*University of Würzburg, Würzburg, Germany*

***To whom correspondence should be addressed:** Dr. Kaija J. Autio, Faculty of Biochemistry and Molecular Medicine, University of Oulu, P.O. Box 5400, FI-90014 University of Oulu, Finland, Tel.: +358 294 48 1142, e-mail: kaija.autio@oulu.fi

Supplementary Figures:

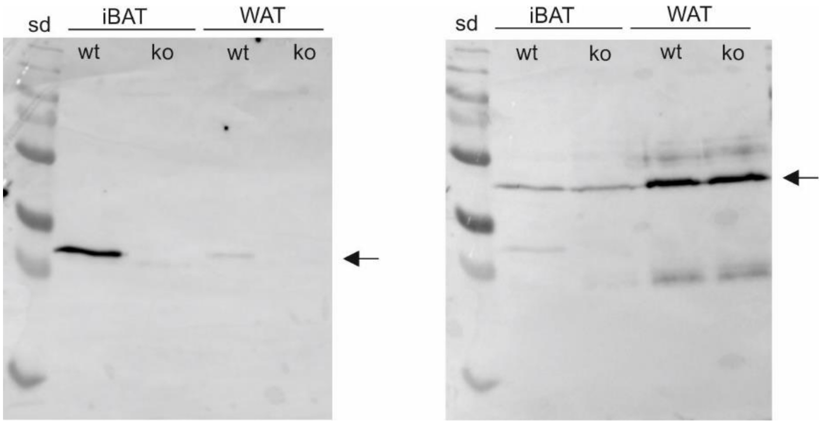

Figure S1. Original Western blots of DECR (left) and β-actin loading control (right) as shown in Figure 3A. Thermo Scientific PageRuler Plus Prestained Protein Ladder (Thermo Fisher Scientific, CA, USA) was used as a molecular size marker (sd).

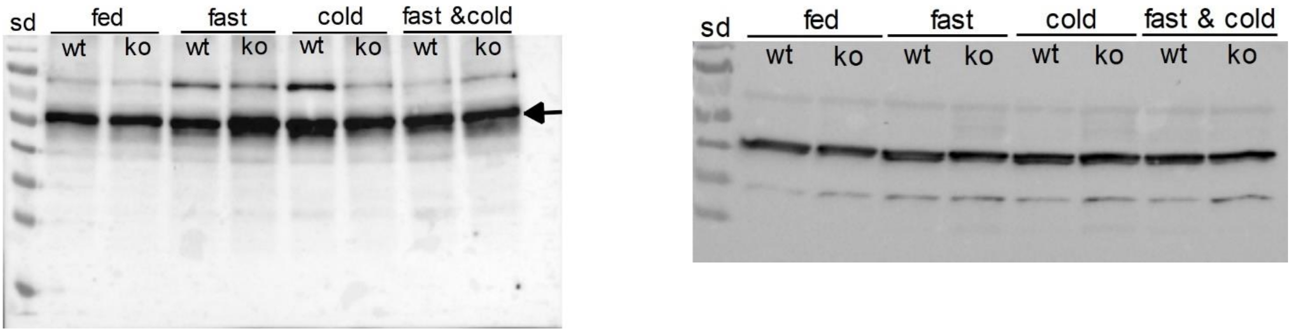

Figure S2. Original Western blots of ATGL (left) and β-actin loading control (right) as shown in Figure 6A. Thermo Scientific PageRuler Plus Prestained Protein Ladder (Thermo Fisher Scientific, CA, USA) was used as a molecular size marker (sd).

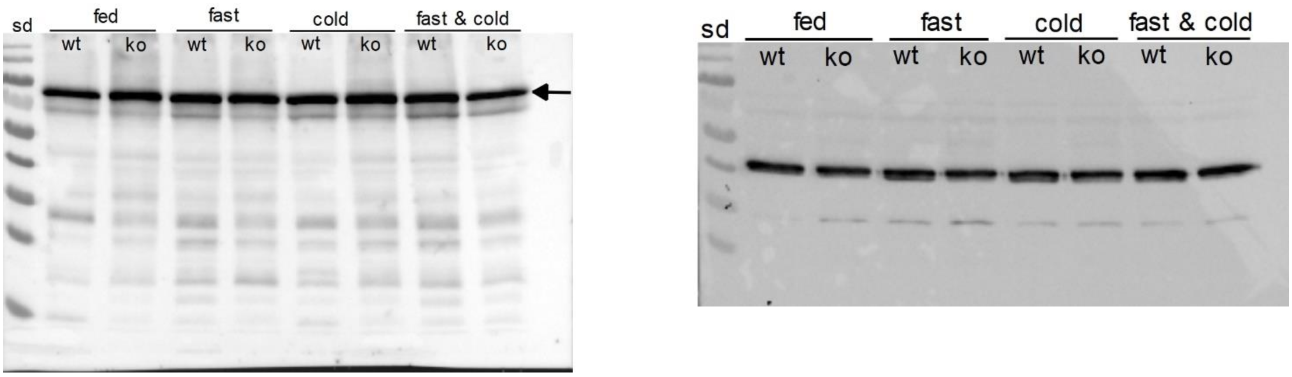

Figure S3. Original Western blots of HSL (left) and β-actin loading control (right) as shown in Figure 6A. Thermo Scientific PageRuler Plus Prestained Protein Ladder (Thermo Fisher Scientific, CA, USA) was used as a molecular size marker (sd).

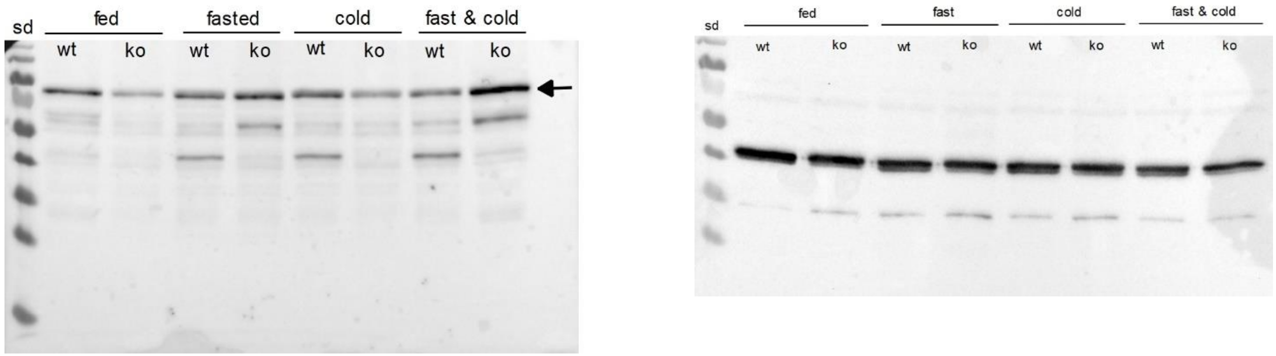

Figure S4. Original Western blots of p-HSL (left) and β-actin loading control (right) as shown in Figure 6A. Thermo Scientific PageRuler Plus Prestained Protein Ladder (Thermo Fisher Scientific, CA, USA) was used as a molecular size marker (sd).

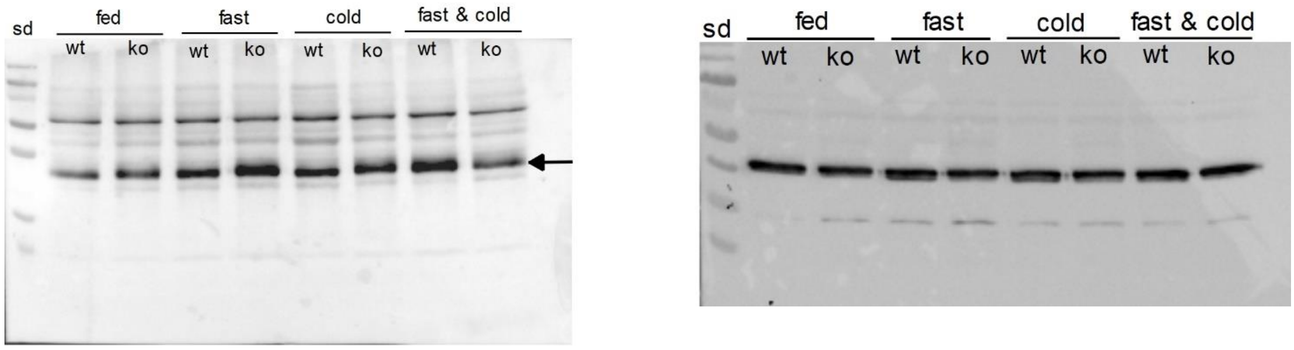

Figure S5. Original Western blots of UCP1 (left) and β-actin loading control (right) as shown in Figure 6A. Thermo Scientific PageRuler Plus Prestained Protein Ladder (Thermo Fisher Scientific, CA, USA) was used as a molecular size marker (sd).

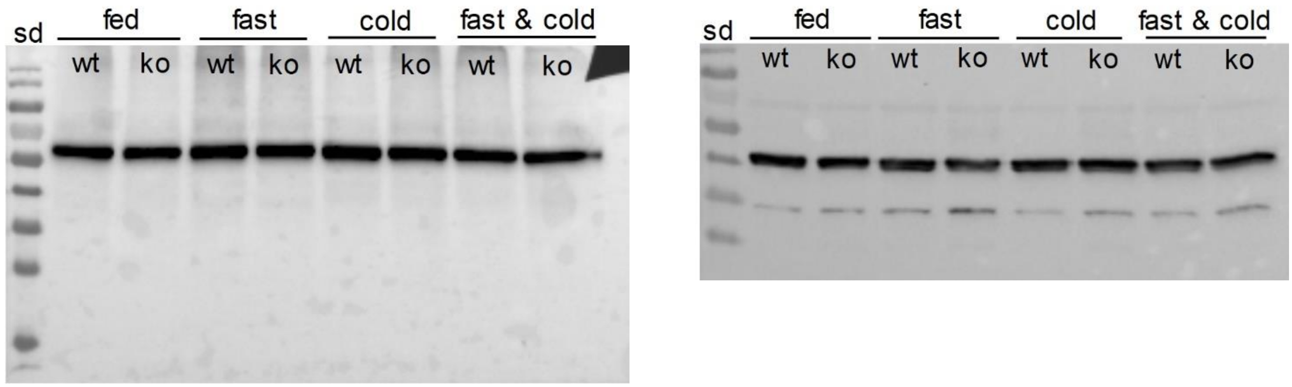

Figure S6. Original Western blots of PKB (left) and β-actin loading control (right) as shown in Figure 6A. Thermo Scientific PageRuler Plus Prestained Protein Ladder (Thermo Fisher Scientific, CA, USA) was used as a molecular size marker (sd).

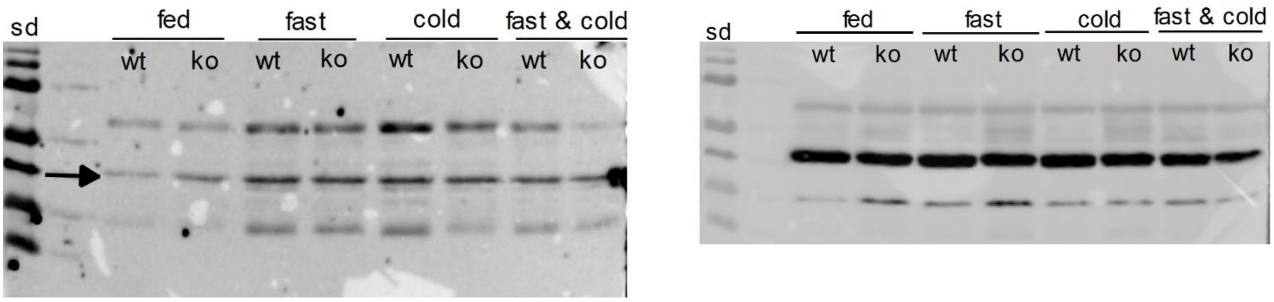

Figure S7. Original Western blots of AMPK (left) and β-actin loading control (right) as shown in Figure 6A. Thermo Scientific PageRuler Plus Prestained Protein Ladder (Thermo Fisher Scientific, CA, USA) was used as a molecular size marker (sd).

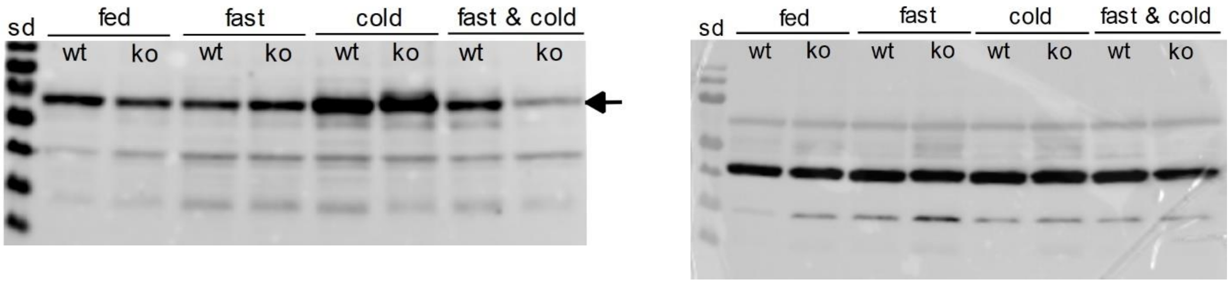

Figure S8. Original Western blots of p-AMPK (left) and β-actin loading control (right) as shown in Figure 6A. Thermo Scientific PageRuler Plus Prestained Protein Ladder (Thermo Fisher Scientific, CA, USA) was used as a molecular size marker (sd).

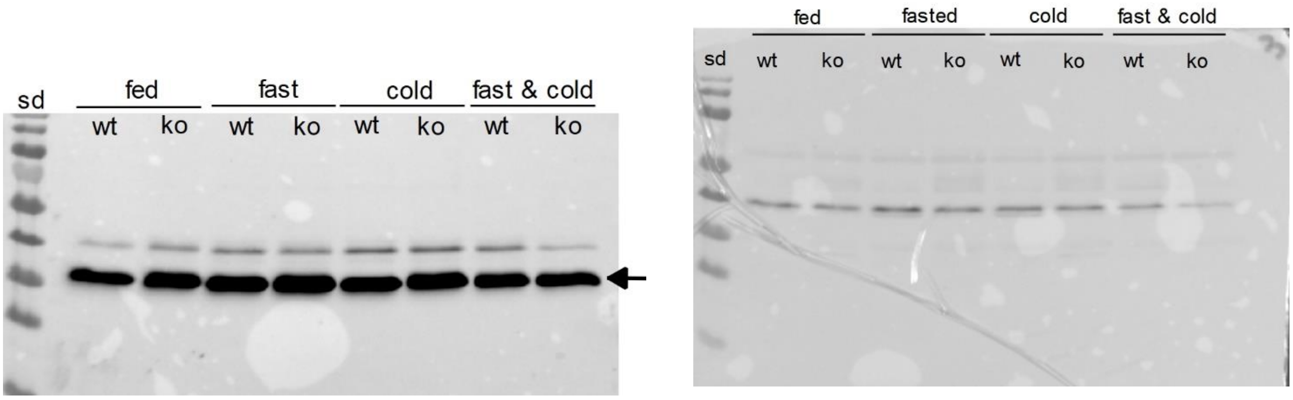

Figure S9. Original Western blots of p38 MAPK (left) and β-actin loading control (right) as shown in Figure 6A. Thermo Scientific PageRuler Plus Prestained Protein Ladder (Thermo Fisher Scientific, CA, USA) was used as a molecular size marker (sd).

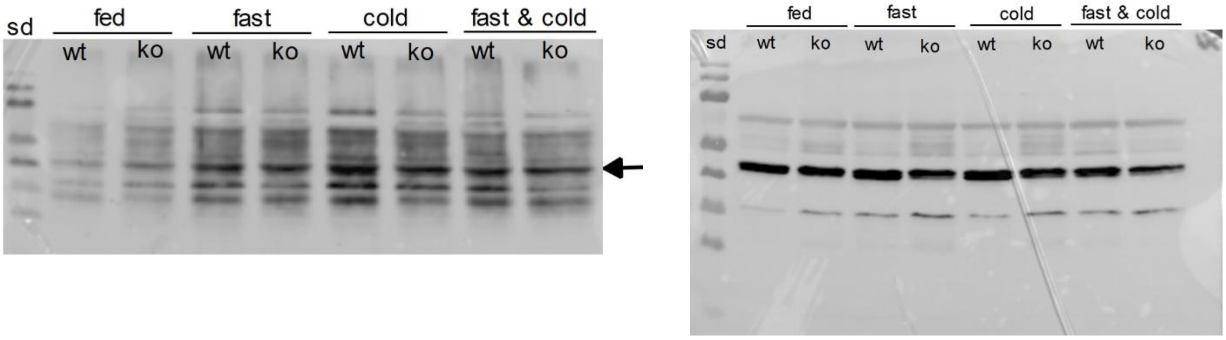

Figure S10. Original Western blots of p-p38 MAPK (left) and β-actin loading control (right) as shown in Figure 6A. Thermo Scientific PageRuler Plus Prestained Protein Ladder (Thermo Fisher Scientific, CA, USA) was used as a molecular size marker (sd).

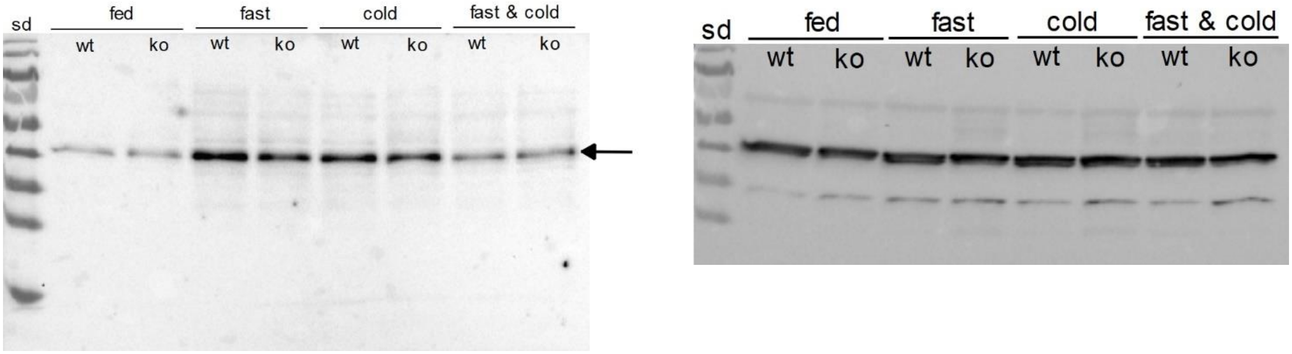

Figure S11. Original Western blots of CREB (left) and β-actin loading control (right) as shown in Figure 6A. Thermo Scientific PageRuler Plus Prestained Protein Ladder (Thermo Fisher Scientific, CA, USA) was used as a molecular size marker (sd).

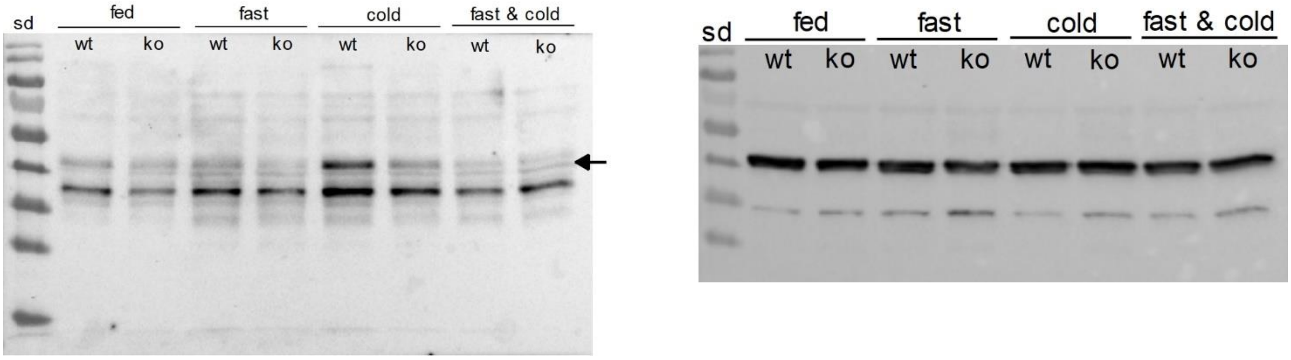

Figure S12. Original Western blots of p-CREB (left) and β-actin loading control (right) as shown in Figure 6A. Thermo Scientific PageRuler Plus Prestained Protein Ladder (Thermo Fisher Scientific, CA, USA) was used as a molecular size marker (sd).

Supplementary Tables:

Supplementary Table S1. Commercial antibodies used in immunoblotting

**Primary Antibodies**	**Manufacturer**	**Catalog number**
Akt/PKB (pan) Rabbit monoclonal antibody (mAb)	Cell Signaling Technology, USA	4691P
AMPKα Rabbit pAb	Cell Signaling Technology, USA	2603
Phospho-AMPKα (Thr172) Rabbit mAb	Cell Signaling Technology, USA	2535
ATGL Rabbit pAb	Cell Signaling Technology, USA	2138
CREB Rabbit mAb	Cell Signaling Technology, USA	9197
Phospho-CREB (Ser133) Rabbit mAb	Cell Signaling Technology, USA	9198
HSL Rabbit pAb	Cell Signaling Technology, USA	4107
Phospho-HSL (Ser660) Rabbit pAb	Cell Signaling Technology, USA	4126
p38 MAP Kinase Rabbit pAb	Cell Signaling Technology, USA	9212
Phospho-p38 (Thr180/Tyr182) Rabbit pAb	Cell Signaling Technology, USA	9211
UCP1 Goat pAb	Novus Biologicals, USA	NB-100-2828
**Secondary Antibodies**	**Manufacturer**	**Catalog number**
Rabbit Anti-Goat-HRP	Santa Cruz Biotechnology, USA	sc-2922
Goat Anti-Guinea pig IgG(H&L)-HRP	Abcam, USA	ab6908
Goat Anti-Rabbit IgG-HRP	Santa Cruz Biotechnology, USA	sc-2004
Goat Anti-Mouse IgG-HRP	Thermo Fisher Scientific, CA, USA	62-6520

Supplementary Table S2. TaqMan Gene Expression Assays

TaqMan Gene Expression Assay	Manufacturer: Thermo Fisher Scientific, CA, USA<br>Catalog number
Atgl/Pnpla2 (adipose triacylglycerol lipase)	Mm00503040_m1
Cox2 (cyclo-oxygenase 2)	Mm03294838_g1
Dio2 (type II iodothyronine deiodinase)	Mm00515664_m1
Elovl3 (fatty acid elongase 2)	Mm00468164_m1
Fgf21 (fibroblast growth factor 21)	Mm00840165_g1
Pgc1a (PPARγ coactivator 1α)	Mm01208835_m1
Ucp1 (uncoupling protein 1)	Mm01244861_m1
